# Childhood adversity is linked to differential brain volumes in adolescents with alcohol use disorder: a voxel-based morphometry study

**DOI:** 10.1007/s11011-014-9489-4

**Published:** 2014-02-05

**Authors:** Samantha J. Brooks, Shareefa Dalvie, Natalie L. Cuzen, Valerie Cardenas, George Fein, Dan J. Stein

**Affiliations:** University of Cape Town, Cape Town, Western Cape South Africa

**Keywords:** VBM, MRI, Alcohol use disorder, Childhood trauma

## Abstract

Previous neuroimaging studies link both alcohol use disorder (AUD) and early adversity to neurobiological differences in the adult brain. However, the association between AUD and childhood adversity and effects on the developing adolescent brain are less clear, due in part to the confound of psychiatric comorbidity. Here we examine early life adversity and its association with brain volume in a unique sample of 116 South African adolescents (aged 12–16) with AUD but without psychiatric comorbidity. Participants were 58 adolescents with DSM-IV alcohol dependence and with no other psychiatric comorbidities, and 58 age-, gender- and protocol-matched light/non-drinking controls (HC). Assessments included the Childhood Trauma Questionnaire (CTQ). MR images were acquired on a 3T Siemens Magnetom Allegra scanner. Volumes of global and regional structures were estimated using SPM8 Voxel Based Morphometry (VBM), with analysis of covariance (ANCOVA) and regression analyses. In whole brain ANCOVA analyses, a main effect of group when examining the AUD effect after covarying out CTQ was observed on brain volume in bilateral superior temporal gyrus. Subsequent regression analyses to examine how childhood trauma scores are linked to brain volumes in the total cohort revealed a negative correlation in the left hippocampus and right precentral gyrus. Furthermore, bilateral (but most significantly left) hippocampal volume was negatively associated with sub-scores on the CTQ in the total cohort. These findings support our view that some alterations found in brain volumes in studies of adolescent AUD may reflect the impact of confounding factors such as psychiatric comorbidity rather than the effects of alcohol per se. In particular, early life adversity may influence the developing adolescent brain in specific brain regions, such as the hippocampus.

## Introduction

Childhood adversity poses a serious risk to the physical, emotional and psychological well-being of young people, often prompting other negative behavior such as substance abuse disorders, including long-term alcohol abuse (Dube et al. 2006; Fetzner et al. 2011; Magnusson et al. 2011; Nayak et al. 2012; Pilowsky et al. 2009; Tucci et al. 2010; Widom et al. 2013; Wu et al. 2010). In the Cape Town wine-producing region of South Africa a high prevalence of adolescent alcohol use disorder (AUD) exists, with alcohol being one of the most commonly abused substances among the young in this region (Parry et al. 2011; Pluddemann et al. 2004), perhaps especially in those with a history of early childhood adversity (Skala and Walter 2013).

Structural and functional brain differences have been observed in adults and adolescents who consume large quantities of alcohol, in brain regions such as the temporal, parietal and occipital lobes, including the hippocampus, as well as frontal lobe deficits (Fein and McGillivray 2007; Fein et al. 2009; Pfefferbaum et al. 1992; Sullivan et al. 1995, 2000, 2010). However, susceptibility to the neurodevelopmental effects of AUD may differ in the adolescent, as compared to the adult brain (De Bellis et al. 2000, 2005; Grant and Dawson 1997; McCarty et al. 2004; Medina et al. 2007; Nagel et al. 2005; Tapert and Schweinsburg 2005). Adolescent neural development, for example, is marked by maturation and selective synaptic pruning in the prefrontal cortex, amygdala and hippocampus, regions which may be particularly susceptible to the effects of alcohol and other substance abuse (Crews et al. 2007; Lenroot and Giedd 2006).

Comorbidity may however confound findings as several psychiatric conditions are also associated with volumetric alterations. Our recent voxel-based morphometric (VBM) study of adolescent AUD focused on a group of adolescents with substance use and without psychiatric comorbidities that may impact on the developing brain (e.g. other substance abuse, conduct, oppositional or defiant disorder). In AUD subjects compared to controls, smaller gray matter volumes were observed in left lateral frontal, temporal and parietal regions (Fein et al. 2013). In our previous study, however, we did not examine the impact of childhood adversity on whole brain volumes, which may explain further variance in the developing brain of an adolescent.

Thus, a range of data indicate the potential impact of early life adversity on the brain, particularly in the prefrontal cortex, anterior cingulate, hippocampus and cerebellum (Tomoda et al. 2009; Kitayama et al. 2006; Carrion et al. 2007; Weniger et al. 2008). More recent brain imaging studies of traumatized individuals have also controlled for common psychiatric comorbidities to differentiate between the effects of psychiatric conditions such as anxiety, depression and childhood adversity (Hanson et al. 2012; De Brito et al. 2013; Teicher et al. 2012).

In the present voxel-based morphometry study (VBM), we extended our analysis of a cohort of adolescents from the Cape Town area of South Africa, with no other psychiatric comorbidities or other substance abuse, to focus in more detail on the link between childhood adversity, AUD and structural brain abnormalities. In our earlier study (Fein et al. 2013) an initial preliminary correlation analysis of early exposure to trauma and brain volumes revealed no significant findings, perhaps due to the region of interest approach taken. It could be that other brain regions, not included in our previous analysis of regions highly implicated in AUD, are associated with the effects of childhood trauma. Thus, here we undertook a different ANCOVA and linear regression approach, allowing us to investigate the contributory roles of both trauma and AUD. We hypothesized that childhood trauma would interact with brain volumes in prefrontal, parietal, temporal and limbic regions.

## Methods

### Participants

See Table 1 for demographics of the total sample. 58 healthy control adolescents and 58 AUD adolescents were matched for age, gender and imaging protocol (more details of the two imaging protocols under Magnetic resonance image acquisition). Thus, constituting a total group of 116 English- and Afrikaans-speaking adolescents who were recruited from 19 schools within the Cape Flats region of the greater Cape Town area. All participants were entirely ‘Cape Coloured’ and from moderately low socioeconomic backgrounds, residing in permanent housing with potable water and electricity, but mostly without luxury items such as computers and cars. Heavy drinking is prevalent across all participants in this socio-economic and geographical cohort, and thus it is likely that the sample were exposed to moderate levels of prenatal alcohol consumption (although it is a limitation that we did not explicitly measure fetal alcohol syndrome in the sample). The median gross annual income level per household was ZAR 62 035. The mean age of the entire sample was 14.85 years (±0.76), and subjects had completed 7.82 years (±0.81) of education and we had fewer females (*n* = 42, 36 %) to males (*n* = 74, 64 %). . Alcohol intake was measured in all participants by using the revised version of the Timeline Followback (TLFB) procedure (Sobell and Sobell 1992). Alcohol life dose was measured in units. One unit was defined as one beer or wine cooler, one glass of wine, or one 1.5-oz shot of liquor (alone or in a mixed drink). Our sample consisted of 1) 58 *alcohol use disorder* (AUD) adolescents who were currently drinking alcohol, and 2) 58 *healthy control* (HC) adolescents who had either never consumed alcohol, or who were light drinkers with a lifetime dosage not exceeding 76 units of alcohol, all with no history of an AUD. In our adolescent sample, the mean number of years that the AUD group had been drinking was 2 years (maximum 6 years), and the mean number of days per month that they drink was 5 (maximum 25 days). AUD and HC were matched according to age and gender.

**Table 1:** Demographics between axial and sagittal protocols with subjects that have had both MRI scans, matched for age and gender

Mean, (s.d.) (unless otherwise stated)	Axial protocol			Sagittal protocol			Effect sizes				
	Total (*n* = 50)	Alcohol (*n* = 25)	Healthy (*n* = 25)	Total (*n* = 66)	Alcohol (*n* = 33)	Healthy (*n* = 33)	Between protocol (total)	Between protocol (alcohol)	Between protocol (healthy)	Alcohol v healthy (axial)	Alcohol v healthy (sagittal)
Age	14.9 (0.9)	15.0 (0.9)	14.7 (0.9)	14.8 (0.7)	14.8 (0.7)	14.8 (0.7)	0.13	0.26	0.13	0.34	0
Gender (m/f)	30/20	15/10	15/10	20/46	10/23	10/23	–	–	–	–	–
Gray matter volume (ml)	766 (82)	754 (81)	779 (82)	779 (74)	779 (68)	769 (80)	0.18	0.34	0.13	0.31	0.14
White matter volume (ml)	426 (48)	431 (53)	421 (42)	421 (45)	426 (46)	415 (44)	0.11	0.1	0.14	0.21	0.25
CSF volume (ml)	131 (110)	113 (24)	149 (154)	120 (30)	115 (32)	119 (27)	0.15	0.07	0.3	0.33	0.14
Total matter volume (ml)	1,192 (120)	1,185 (122)	1,200 (119)	1,200 (110)	1,204 (103)	1,183 (117)	0.07	0.17	0.15	0.13	0.19
Left STG (ml)^a^	8.3 (1.0)	8.3 (1.1)	8.4 (0.9)	8.5 (0.8)	8.6 (0.7)	8.4 (0.9)	0.23	0.34	0	0.1	0.25
Right STG (ml)^a^	6.5 (0.9)	6.4 (0.9)	6.6 (0.8)	6.5 (0.7)	6.5 (0.6)	6.4 (0.8)	0	0.14	0.25	0.24	0.14
Left hippocampus (ml)^a^	3.2 (0.5)	3.2 (0.5)	3.2 (0.4)	3.4 (0.4)	3.3 (0.4)	3.3 (0.4)	0.45	0.23	0.25	0	0
Right hippocampus (ml)^a^	3.3 (0.4)	3.3 (0.4)	3.3 (0.4)	3.3 (0.4)	3.3 (0.5)	3.3 (0.4)	0	0	0	0	0
Alcohol life dose (units^b^)	990 (1,532)	1,975 (1,663)	5 (8)	496 (865)	985 (1,013)	5 (17)	0.42	0.76	0	1.71	1.39
CTQ: *Physical neglect*	9 (3.9)	9 (4)	9 (4)	8 (3)	8 (3)	8 (3)	0.3	0.29	0.29	0	0
CTQ: *Emotional neglect*	15 (6.4)	14 (6)	15 (7)	11 (6)	11 (6)	11 (5)	0.65	0.51	0.69	0.16	0
CTQ: *Physical abuse*	7 (4)	7 (4)	7 (4)	6 (2)	7 (3)	6 (1)	0.33	0	0.37	0	0.45
CTQ: *Emotional abuse*	8 (4)	9 (4)	8 (4)	8 (4)	9 (3)	8 (4)	0	0	0	0.26	0.29
CTQ: *Sexual abuse*	7 (3)	7 (4)	6 (2)	6 (3)	6 (3)	6 (2)	0.34	0.29	0	0.32	0
CTQ: *Total score*	46 (14.9)	46 (16)	45 (13)	38 (12)	41 (14)	38 (10)	0.61	0.34	0.63	0.07	0.25

^a^Estimates based on percentage of MNI aal template

^b^Alcohol life dose was measured in units. One unit was defined as one beer or wine cooler, one glass of wine, or one 1.5-oz shot of liquor (alone or in a mixed drink)

Participants were screened for eligibility after written informed assent/consent was obtained from volunteers and parents or guardians. Screening involved detailed medical history-taking, physical and psychiatric examination, and urine analysis and breathalyzer testing (to confirm that the adolescents were not intoxicated during the testing procedures), all performed by a fully qualified and licensed psychiatrist. The Schedule for Affective Disorders and Schizophrenia for School Aged Children (6–18 years) Lifetime Version (K-SADS-PL) (Kaufman et al. 1996), a semi-structured clinician-rated diagnostic scale, was used to ascertain current and past psychiatric diagnoses, as reported by the participants.

Exclusion criteria for study participation were: mental retardation, lifetime DSM-IV Axis I diagnoses other than AUD (including depressive, anxiety, psychotic, post-traumatic stress, elimination, eating, tic, attention-deficit/hyperactivity, oppositional defiant, and conduct disorders); lifetime dosages exceeding 30 cannabis joints or 3 methamphetamine doses; current use of sedative or psychotropic medication; signs or history of or malnutrition (using physical examination, for example, of head circumference, joints and muscles, bone length); sensory impairment; history of traumatic brain injury with loss of consciousness exceeding 10 min; presence of diseases that may affect the CNS (e.g., meningitis, epilepsy, HIV); less than 6 years of formal education; and lack of proficiency in English or Afrikaans. Collateral information verifying the absence of medical, psychiatric, and psychosocial problems was obtained from consenting parents by a social worker. The social worker consulted with schoolteachers at pre-screening interviews to confirm whether participants’ behavior and performance at school were considered to be within age-appropriate parameters. This information was not recorded, but merely used to assess the eligibility of our final recruitment. To assist the participants in completing the self-report demographic and early adversity questionnaires, a research assistant was present at all times. All test materials were available in the participant’s language of preference.

Meals and refreshments were provided for participants, and study participation was compensated by gift vouchers (to the value of ZAR 50 per visit), distributed at the conclusion of the testing session. All study information was kept confidential, except where statutory requirements dictated the reporting of newly identified or ongoing threats to the safety of minor participants. The study protocol and procedures complied with and were conducted in strict adherence to the guidelines contained in the Declaration of Helsinki (2008). Full written approval to conduct the study was obtained from the Western Cape Education Department and the Research Ethics Committee of the Stellenbosch University Faculty of Health Sciences.

### Measures

#### Substance use

A revised version of the Timeline Followback (TLFB) procedure (Sobell and Sobell 1992), a semi-structured, clinician-administered assessment of lifetime history of alcohol use and drinking patterns, was used in collaboration with the K-SADS-PL to elicit alcohol-use data.

#### Early adversity

We used the Childhood Trauma Questionnaire-Short Form (CTQ-SF; Bernstein et al. 2003) to measure early adversity. The CTQ-SF is a 28-item retrospective self-report questionnaire comprising five subscales, each of which is aimed at measuring a distinct dimension of childhood mistreatment: physical abuse, sexual abuse, emotional abuse, physical neglect, and emotional neglect. Each type of maltreatment is represented by five items. An additional three-item minimization/denial subscale is included to detect the under-reporting of maltreatment. Item response options are structured to reflect the frequency of maltreatment experiences (i.e., never true, rarely true, sometimes true, often true, very often true), and are scored from 1 to 5 accordingly. Each of the five primary subscales has demonstrated good internal consistency across a range of heterogeneous samples, including individuals with SUDs (physical abuse subscale = 0.83 to 0.86; sexual abuse = 0.92 to 0.95; emotional abuse = 0.84 to 0.89; physical neglect = 0.61 to 0.78; emotional neglect = 0.85 to 0.91). In our sample, the internal consistency for the CTQ was overall 0.77 (physical abuse subscale = 0.74; sexual abuse = 0.76; emotional abuse = 0.75; physical neglect = 0.73; emotional neglect = 0.73).

### Magnetic resonance image acquisition

All MRIs were collected on a 3T Siemens Magnetom Allegra MR Headscanner using Syngo MR software. The scanner is located in the Cape Universities Brain Imaging Center at the Stellenbosch University Health Sciences Campus, Tygerberg Hospital, South Africa. Fifty subjects were imaged using a transaxial T1-weighted acquisition (TR = 2,080 ms, TE = 4.88 mm, acquisition matrix = 256 × 192) at 1.0 mm thickness. The initial review of these images revealed undesirable presence of blood-vessels in the imaging, resulting from the fact the scanner used is a head-only model. This does not allow proper saturation of the blood to suppress its signal before it enters the head. To reduce the signal from unsaturated blood, the use of a sagittal T1 protocol was instituted (TR = 2,200 ms, TE = 5.16 ms, acquisition matrix 256 × 256) at 1.0 mm thickness. Twenty-eight subjects were imaged using both protocols, in total, 66 subjects were imaged using the sagittal protocol. In previous work (Fein et al. 2013), for participants who were studied with both MRI acquisition protocols, we estimated GM and subcortical volumes from both images and computed the Pearson’s correlation to assess the comparability of GM segmentations. All correlation coefficients were significant (*p* < 0.002) and ranged from *r* = 0.632 to 0.993.

### MRI image analysis

All images were rotated/reoriented into the AC-PC plane in all our nifti-coverted DICOM T1 images, and initial quality control for signal artifacts, morphological changes were calculated in gray matter by segmenting from white matter and cerebrospinal fluid using the voxel-based morphometry (VBM) unified segmentation approach (Ashburner and Friston 2000) in SPM8 (www.fil.ucl.acuk/spm8). Following this segmentation procedure, probability maps of gray matter were “modulated” to account for the effect of spatial normalization to the template during segmentation. We did this as per the default settings in SPM8, by multiplying the probability value of each voxel by its relative volume in native space before warping. Gray matter images based on probability values at each voxel were spatially normalised using a pediatric template for 12–16 year old children from the Cincinnati Children’s Hospital old children template (www. irc.cchmc.org/software/pedbrain.php) and then coregistered using the same segmented template. Modulated images were smoothed with an 8 mm ‘Full Width Half Maximum [FWHM]’ Gaussian kernel, consistent with other recent VBM studies. This smoothing kernel was applied prior to statistical analysis, to reduce signal noise and to correct for image misregistration.

### VBM analyses

#### Full factorial 2 × 2 ANCOVA (Group × Protocol with CTQ as covariate of interest)

In the total cohort, to examine the main effects of group (AUD and HC) and of protocol (axial and sagittal acquisition) on brain volume data, we conducted a 2 × 2 ANCOVA. We added age, gender and total matter volume (we did not use total intracranial volume, consisting of the sum of gray matter, white matter and cerebrospinal fluid, but instead only used gray and white matter volume data, because SPM may over-estimate cerebrospinal fluid) as covariates of no interest, and total CTQ score as covariate of interest.

#### Within-protocol regression analyses with CTQ

Using a VBM multiple regression approach, we built a basic model controlling for age, gender, total alcohol intake and total matter (gray and white) volume. With the basic model, we calculated a multiple regression to model positive and negative associations between brain volume and total CTQ score across all subjects. We then re-ran the same model to examine regressions with CTQ in the AUD and HC groups separately.

#### Correlations between brain volumes, CTQ and lifetime alcohol intake

Using MarsBaR (www.http://marsbar.sourceforge.net) we extracted Automated Anatomical Labeling (AAL, Tzourio-Mazoyer et al. 2002) brain volumes post-hoc for bilateral regions of interest that showed as differential in our previous analyses, including: bilateral superior temporal gyrus, bilateral precentral gyrus and bilateral hippocampus. For the demographics table, we converted morphological data for these regions, and global gray, white and CSF volumes, in to ml values using ml values for each of the AAl regions and multiplying by the extracted data from our sample (personal communication, Dr. Matthew Kempton, King’s College, London). The total sample was correlated with total and subscales of CTQ and lifetime alcohol intake using SPSS (http://www-01.ibm.com/software/analytics/spss/). For the correlation analyses we used Pearson’s parametric coefficient after examining the data for normal distribution and converting to z-scores. See Table 2 for details of brain volume data for these Regions of Interest.

**Table 2:** Demographics of the total group combined (matched for protocol, age and gender)

Mean, (s.d.) (unless otherwise stated)	Total group (*n* = 116)			
	Alcohol (*n* = 58)	Healthy (*n* = 58)	Effect size	*P* value
Age	14.9 (0.8)	14.7 (0.8)	0.25	n.s.
Gender (m/f)	25/33	25/33	–	–
Home language				
English	12	10	–	–
Afrikaans	41	45	–	–
Bilingual	5	3	–	–
Gray matter volume (ml)	768 (74)	779 (80)	0.14	n.s.
White matter volume (ml)	428 (49)	418 (43)	0.22	n.s.
CSF volume (ml)	114 (28)	136 (102)	0.3	n.s.
Total matter volume (ml)	1,196 (111)	1,197 (117)	0.01	n.s.
Left STG (ml)^a^	8.4 (0.9)	8.4 (0.8)	0.0	n.s.
Right STG (ml)^a^	11.3 (1.3)	11.4 (1.2)	0.08	n.s.
Left hippocampus (ml)^a^	3.2 (0.4)	3.3 (0.4)	0.25	n.s.
Right hippocampus (ml)^a^	3.3 (0.4)	3.4 (0.4)	0.25	n.s.
Alcohol life dose (units^b^)	1,412 (1,409)	6 (14)	**1.42**	<**0.001**
No. of smokers	33	4	–	–
No. of years drinking	2 (1)	0	–	–
No. of drinking days per month	5 (4)	0	–	–
No. of average drinks per month	15 (4.5)	0	–	–
CTQ: *Physical neglect*	9 (3)	8 (4)	0.29	n.s.
CTQ: *Emotional neglect*	12 (6)	13 (6)	0.17	n.s.
CTQ: *Physical abuse*	7 (3)	6 (3)	0.34	n.s.
CTQ: *Emotional abuse*	9 (4)	7 (4)	**0.5**	**0.09**
CTQ: *Sexual abuse*	7 (4)	6 (2)	0.32	n.s.
CTQ: *Total score*	43 (15)	40 (12)	0.22	n.s.

*n.s.* non significant, *ml* millilitres, *STG* superior temporal gyrus, *CTQ* Childhood Trauma Questionnaire

^a^Estimates based on percentage of MNI aal template

^b^Alcohol life dose was measured in units. One unit was defined as one beer or wine cooler, one glass of wine, or one 1.5-oz shot of liquor (alone or in a mixed drink)

*Bolded* values represent significant differences

## Results

### Psychometric data

In the total cohort, as expected, adolescents with AUD had significantly higher levels of lifetime alcohol intake than healthy adolescents (large effect size: 1.42), as well as a trend to higher levels of self-reported emotional abuse (compared to other types of abuse and neglect) as measured by the CTQ (medium effect size: 0.5).

### Full factorial 2 × 2 ANCOVA (Group × Protocol with CTQ as covariate of interest)

**Fig. 1:**
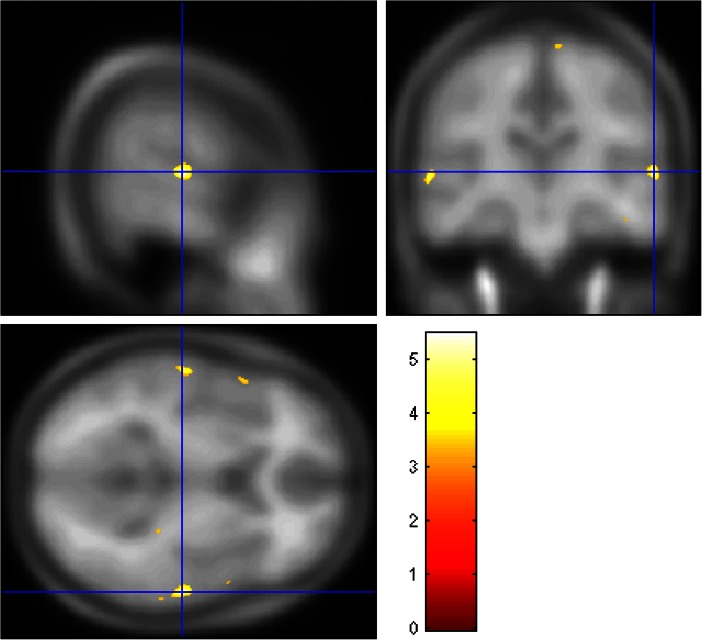
Reduced brain volume in the AUD compared to the HC group, overlaid onto the Cincinnati Children’s hospital 12–16 year old template for accuracy, in the superior temporal gyrus as shown in both the ANCOVA main effect of group, and post-hoc t-tests, correcting for age, gender, total matter volume. *Heat bar* represents F-statistic

See Table 3. A peak FWE corrected significant morphological estimation main effect of group (with total CTQ score as covariate) was found in the right superior temporal gyrus (*x* = 64, *y* = −24, *z* = 100, *p* = 0.05_FWE peak corrected_)_._ When CTQ was removed as a covariate of interest, the main effect included bilateral superior temporal gyrus (*x* = −65, *y* = −18, *z* = 8). Post-hoc t-tests revealed that there were no greater regions of brain volume in the AUD compared to the HC group. However, it was confirmed that the bilateral superior temporal gyrus coordinates reported above were reduced in AUD compared to HC. (See Fig. 1).

**Table 3:** 2 × 2 ANCOVA in groups matched for age and gender (Group × Protocol with CTQ as covariate of interest, total matter volume as covariates of no interest) and post-hoc t-tests

Brain region	MNI coordinates			Cluster size (voxels)	Peak FWE corrected
	*x*	*y*	*z*		
Main effect of GROUP (with CTQ)					
Right superior temporal gyrus	64	−21	10	224	0.05
Main effect of GROUP (without CTQ)					
Right superior temporal gyrus	64	−21	10	338	0.01
Left superior temporal gyrus	−65	−18	8	281	0.05
Main effect of PROTOCOL					
Left uncus/temporal lobe/rectal gyrus	−23	2	−32	34,339	0.001
Cerebellum	0	−43	−57		
Interactions					
Left uncus/temporal lobe/rectal gyrus	−23	2	−32	4,093	0.001
Alcohol > healthy (with/without CTQ)					
–	–	–	–	–	–
Healthy > alcohol (with CTQ)					
Right superior temporal gyrus	64	−21	10	424	0.008
Left superior temporal gyrus	−65	−18	8	373	0.03
Healthy > alcohol (without CTQ)					
Right superior temporal gyrus	64	−21	10	431	0.008
Left superior temporal gyrus	−65	−18	8	381	0.03

*CTQ* Childhood Trauma Questionnaire, *MNI* Montreal Neurological Institute Coordinates, *FWE* Family Wise Error corrected for multiple comparison (at the peak voxel level)

### Total group regression analyses with CTQ

**Fig. 2:**
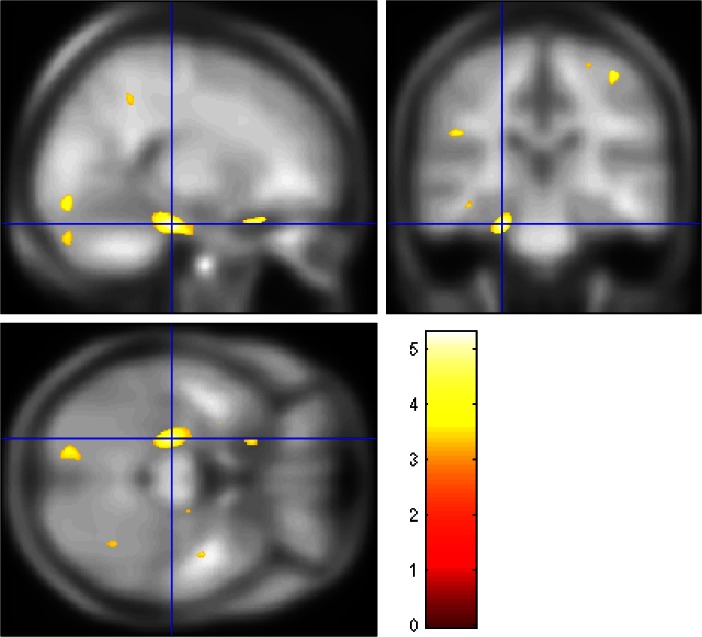
Reduced brain volume associated with higher total CTQ score, overlaid onto the Cincinnati Children’s hospital 12–16 year old template for accuracy, in the left hippocampus, as shown in the regression analysis controlling for protocol, age, gender, lifetime alcohol intake and total matter volume. *Heat bar* represents F-statistic

See Table 4. Given that there was no significant difference between the total AUD and HC groups in total CTQ score and total matter volume (see Table 3), we ran a regression analysis to examine the effect of total CTQ on brain volume in the total cohort. Examining the total cohort together (controlling for protocol, age, gender, lifetime alcohol intake and total matter volume) for associations between estimated brain volumes and total CTQ score, we found no positive correlations, and only negative correlations in the right precentral gyrus (*x* = 42, *y* = −23, *z* = 65), *p* = 0.03_FWE corrected_ and left hippocampus (*x* = −24, *y* = −27, *z* = −21) *p* = 0.05_FWE corrected._ (See Fig. 2).

**Table 4:** Multiple regression in total group with total CTQ score as covariate of interest, and age, gender, total matter volume and protocol as covariates of no interest

Positive CTQ regression (high total score associated with larger BV)					
Total group					
–	–	–	–	–	–
AUD group					
–	–	–	–	–	–
HC group					
–	–	–	–	–	–
Negative CTQ regression (high total score associated with smaller BV)					
Total group					
Right precentral gyrus	42	−23	65	697	0.03
Left hippocampus	−24	−27	−21	1838	0.05
AUD group					
–	–	–	–	–	–
HC group					
–	–	–	–	–	–

All analyses corrected for age, gender and total matter volume

Repeating the regression analyses in the AUD and HC groups separately revealed no significant correlations between total CTQ and brain volume.

### Correlations between ROI brain volumes, CTQ and lifetime alcohol intake in total cohort

See Table 5. Given that we found significant regressions between brain volumes and total CTQ in the superior temporal gyrus, hippocampus and precentral gyrus we extracted bilateral brain volume data from these regions using the AAL atlas with MaRsBAr and correlated with CTQ total and subscales, as well as with lifetime alcohol intake. CTQ total score also positively correlated with lifetime alcohol intake (Rho 0.20, *p* = 0.05). Further, left hippocampal volume negatively correlated with CTQ scores, specifically physical neglect (Rho −0.22, *p* = 0.01), emotional neglect (Rho −0.19, *p* = 0.01), physical abuse (Rho −0.21, p0.01), emotional abuse (Rho −0.23, p0.01), sexual abuse (Rho −0.23, p0.01) and total CTQ (Rho −0.31, *p* = 0.01). Right hippocampal volumes also correlated with CTQ scores, namely, emotional neglect (Rho −0.16, *p* = 0.01), sexual abuse (Rho −0.16, *p* = 0.01) and total CTQ score (Rho −0.21, *p* = 0.01). No CTQ scores correlated with any other brain regions.

**Table 5:** Significant pearson coefficients between brain volumes and CTQ subscale scores

	PN	EN	PA	EA	SA	Tot
LAI	0.19*		0.20*	0.27**	0.19**	0.20*
L_Hc	−0.22**	−0.19**	−0.21**	−0.23**	−0.23**	−0.31**
R_Hc		−0.16**			−0.16**	−0.21**

Childhood Trauma Questionnaire subscales: *PN* physical neglect, *EN* emotional neglect, *PA* physical abuse, *EA* emotional abuse, *SA* sexual abuse, *Total* total CTQ score, *LAI* lifetime alcohol intake, *L* left, *R* right, *Hc* hippocampus, (all coefficient scores represent Z scores, checked for normal distribution, log transformed). No other brain regions (observed as differential in our previous analyses) were significantly correlated with CTQ scores

*p0.05, **p0.01

## Discussion

These data reveal that childhood trauma and alcohol use disorder (AUD) in a cohort of South African adolescents are linked to differences in brain volume in comparison to healthy adolescents. Supporting our previous analyses of AUD (Fein et al. 2013), but now using a full factorial analysis of covariance (ANCOVA), we confirmed that temporal volumes were reduced in adolescents with AUD compared to healthy subjects, although we were not able to replicate reduced brain volumes in the frontal and parietal cortices, and no gender-specific effects on brain, and in addition we found reduced effects in the hippocampi. This may be due, in part, to the different statistical model employed and our addition of CTQ scores here. We did not, however, find an interaction between AUD and CTQ, and only a trend for higher levels of emotional abuse in the AUD cohort compared to healthy controls, suggesting that there are additional factors (e.g. genetic, socio-economic) that influence how CTQ and AUD status in adolescents interact with brain volume. There was some evidence that, a higher CTQ score was associated with volumetric reductions, specifically in right precentral gyrus and bilateral hippocampus. These results were seen even after controlling for AUD, indicating that they are more likely attributable to childhood trauma than adolescent alcohol use per se.

Our most robust finding was that bilateral superior temporal cortices were significantly reduced in the adolescent AUD group compared to healthy controls, and this result survived independently of childhood trauma. These findings, in line with our previously published work (Fein et al. 2013) suggest that the superior temporal lobes are most susceptible to excessive alcohol intake during the developmental period of adolescence. However, ours and other studies have found that fronto-temporal regions are most susceptible to alcoholism in adults (Grodin et al. 2013; Fein et al. 2013), though here we did not find frontal lobe deficits. Temporal lobe susceptibility might lead to Wernicke-Korsakoff syndrome in adolescents who continue to abuse alcohol, a condition typified by memory and comprehension deficits, which are particularly detrimental for school-age individuals.

Our subsequent regression analysis showed that levels of childhood trauma in the total cohort were most significantly linked to reduced volume in the right precentral gyrus and left hippocampus. These findings were not observed in our previous work, but support the view that reductions in hippocampal volumes are associated with other psychiatric/trauma comorbidities, rather than mere alcohol abuse alone, in an AUD adolescent cohort. Other studies of AUD have found reduced hippocampal volumes, but these studies did not take in to account levels of childhood trauma (Nagel et al. 2005; De Bellis et al. 2000, 2005; Medina et al. 2007, 2008), and so it is not known whether the findings of these previous studies were driven by past experience of trauma. It is plausible, given our findings that volumetric differences in the hippocampus may be due to degrees of past and present trauma exposure in those with AUD, rather than to ethanol exposure alone. In support of this, a recent VBM study of traumatized refugees without AUD or other comorbid disorders reported reduced hippocampal volumes (Eckart et al. 2012).

Regression analyses with total CTQ score in the total cohort (controlling for AUD status, age, gender, and total matter volume) revealed that a higher CTQ score was associated with smaller brain volumes in the right precentral gyrus and left hippocampus. Previous evidence has also indicated that smaller bilateral hippocampal volume might be associated with childhood trauma (Samplin et al. 2013), and we also found that the bilateral hippocampus correlated with all childhood trauma scores in our total cohort, independently of AUD status. Other previous studies have also found that AUD is linked to smaller hippocampal volumes (De Bellis et al. 2000, 2005; Medina et al. 2007; Nagel et al. 2005). Specifically, smaller left hippocampal volumes in our total cohort were associated with higher scores on all childhood trauma sub-scales, and smaller right hippocampal volumes were associated with higher scores on emotional neglect, sexual abuse and total childhood trauma score.

An explanation for the link between high childhood trauma scores and reduced brain volume might be that a neural circuit, involving the hippocampus may be involved in the aetiology of adolescent AUD, especially in those exposed to childhood trauma. For example, a recent neuroimaging study reported that reduced hippocampal activity, combined with greater dorsolateral prefrontal cortex activity occurred when anxious patients attempted to suppress uncomfortable emotions (Aybek et al. 2014), and brain function may alter neural plasticity and brain structure over time. Furthermore, functional imaging studies have revealed that inhibitory networks in the brain perform at a reduced level in adolescents with AUD compared to healthy controls (Wetherill et al. 2013), which may also link to evidence that the prefrontal cortex is slow to develop during adolescence (Konrad et al. 2013). Consistent with the view that abnormal brain function in AUD alters brain structure, diffusion weighted imaging has shown that white matter micro-abnormalities may be present in adolescents who abuse alcohol (Baker et al. 2013). However, it must be borne in mind that these differences in brain structure can be either the cause or effect of AUD and the experience of childhood trauma, as the directionality of findings in structural brain imaging studies such as ours is often ambiguous. Nevertheless, early exposure to ethanol may detrimentally affect specific networks in the developing brain associated with inhibitory control, predisposing an adolescent to limited cognitive self-regulation strategies particularly in those who are also exposed to adverse life events early on in life.

Our study has some limitations that should be considered when interpreting these data. We did not collect direct measures of fetal alcohol syndrome disorder (FASD) or amount of prenatal exposure to alcohol, which in this Cape-Coloured, Cape Flats, English/Afrikaans-speaking adolescent cohort is likely to be of high prevalence. For example, facial dysmorphology is the most robust measure of fetal alcohol syndrome in children, and so future studies should use this measure to control for the effects of prenatal exposure to alcohol on brain. Additionally, the reading ability of some of our adolescent cohort may have prevented a strong understanding of some of the self-report measures they were asked to complete, although a research assistant was always present to clarify questions. The total brain volume data in our study was collected using two different protocols (albeit on the same scanner at the same site): axial and sagittal. As the most recent paper from our group documented (Fein et al. 2013), the axial acquisition may cause vasculature artifacts in signal intensity that might be reduced using a sagittal protocol. However, we conducted statistical analyses, both in a small group who had both protocols (*n* = 28), as well as full factorial analyses to examine the main effect of protocol on brain volume measurements. Both approaches showed only minor differences in brain volume measurements, and not in the main regions of differential volume we report.

In conclusion, these findings support our view that some alterations found in brain volumes in studies of adolescent AUD may reflect the impact of confounding factors such as the experience of traumatic life experiences, rather than the effects of alcohol per se. In particular, early life adversity may influence the developing adolescent brain in specific brain regions, such as the hippocampus, but that alcohol exposure alone most significantly affects the temporal lobes in adolescents. Longitudinal studies are needed to determine the impact of both excessive alcohol use and childhood trauma on brain structure over time, and to understand the correlates of such changes with emotional systems such as self-regulation. Future studies could also examine the impact of interactions between early adversity and genetic variation on brain structure in individuals with AUD.
